# Radiostereometric measurement of implant migration in robotically assisted vs conventional bi-cruciate stabilized cemented total knee arthroplasty: secondary analysis of a randomized controlled trial

**DOI:** 10.2340/17453674.2025.43081

**Published:** 2025-03-21

**Authors:** Øystein SKÅDEN, Ove Nord FURNES, Stein Håkon Låstad LYGRE, Irene Ohlen MOLDESTAD, Geir HALLAN, Anne Marie FENSTAD, Paul Johan HØL, Øystein Johannes GØTHESEN

**Affiliations:** 1 Haugesund Hospital for Rheumatic Diseases, Haugesund; 2 Department of Clinical Medicine, University of Bergen, Bergen; 3 Norwegian Arthroplasty Register, Department of Orthopaedic Surgery, Haukeland University Hospital, Helse Bergen HF, Bergen; 4 Biomatlab, Department of Orthopaedic Surgery, Haukeland University Hospital, Helse Bergen HF, Bergen; 5 Department of Occupational Medicine, Haukeland University Hospital, Helse Bergen, Bergen; 6 Coastal Hospital in Hagevik, Helse Bergen, Bergen, Norway

## Abstract

**Background and purpose:**

Robotically assisted computer navigation (robotic) has been developed to improve the positioning in total knee arthroplasties (TKAs), attempting to achieve better functional results and longevity of the prostheses. However, the benefit of robotics is still controversial. The aim of our study was to compare migration between robotic and conventional techniques in cemented bi-cruciate stabilized TKAs, using radiostereometric analysis (RSA) based on a secondary analysis of a randomized controlled trial (RCT).

**Methods:**

We enrolled 60 TKA patients from one hospital (2020–2021), with osteoarthritis or arthritic disease. The patients were examined up to 24 months after the surgery, to estimate the mechanical stability of the tibial component. The maximum total point motion (MTPM) representing the magnitude of migration, the largest negative (subsidence) and positive (lift-off) value for y-translation, and prosthetic rotations were measured. The migration in the 2 groups was compared and the precision evaluated.

**Results:**

51 RSA marked TKAs were available for a comparison of tibial migration between robotically assisted (n = 26) and conventional operations (n = 25). The MTPM in the first year was 0.44 mm and 0.64 mm, and at 24 months 0.46 mm and 0.75 mm, for the conventional and the robotic groups, respectively. The robotic group migrated more than the conventional group at 2 years, 0.21 mm (95% confidence interval [CI] 0.05–0.44; P = 0.01). The overall median MTPM for the investigated implants (both groups) up to 12 months was 0.54 mm (CI 0.44–0.63), and 0.19 mm between 12 and 24 months (CI 0.16–0.22). The magnitude of migration and rotation around the 3 axes was small for both groups, but flexion/extension migration of the tibial component was slightly higher in the robotic group 0.14° (CI 0.00–0.33; P = 0.049).

**Conclusion:**

MTPM and flexion/extension migrations of the tibial component were higher for the robotic group, up to 24 months. The overall migration pattern for the bi-cruciate stabilized implant was acceptable.

Total knee arthroplasty (TKA) is an effective treatment for severe osteoarthritis (OA), with an excellent overall survival rate [1]. However, up to 20% of patients are reportedly dissatisfied due to residual pain, discomfort, and restricted knee function [2]. Computer navigation systems aid in accurate implant positioning and alignment but have not yet shown to improve clinical results and longevity of the implant [3-10]. Robotic computer navigation systems execute bone cuts/burring in the pursuit of even more precise implant fitting than regular computer navigation systems. The downsides are higher costs and increased operating time. Conventional instrumentation is cost effective, with the challenge being repeatable accuracy in component positioning and extremity alignment. Gøthesen et al. [11] showed in a study from the Norwegian Arthroplasty Register (NAR) that the short-term risk of revision was either the same or higher for computer-assisted surgery (CAS) compared with conventional surgery (CONV) and depended more on the brand of the prosthesis. A later study from Dyrhovden et al. [8] with longer follow-up showed similar results for CAS compared with conventional TKAs, but fewer revisions for malalignment for CAS. A bi-cruciate stabilized prosthesis emulating the native anatomy and kinematics of the human knee (Journey II BCS, Smith & Nephew, Memphis, TN, USA) was developed in pursuit of a better functional outcome. This implant has not yet been evaluated through a radiostereometric analysis (RSA). A 10-year survivorship with ODEP (Orthopaedic Data Evaluation Panel) 10 A, or a minimum of 2 years’ RSA evaluation showing safe migration patterns, is advised before use of prosthesis implants in everyday clinical practice in Norway, to avoid using implants susceptible to early aseptic loosening and revision [12]. To our knowledge this is the first RSA analysis comparing a robotic with a conventional technique for TKA.

The aim of our study was to compare migration between robotic and conventional techniques in cemented bi-cruciate stabilized TKA using radiostereometric analysis (RSA).

## Methods

### Study design

This is a study based on a secondary analysis of RSA data collected from the first 60 patients enrolled in a larger randomized controlled trial (RCT). The results from the primary RCT will be reported as 2-year clinical and radiological outcome of robotic versus conventionally operated total knees (214 patients) when follow-up is completed.

The trial was designed and conducted according to the CONSORT statement guidelines for reporting of parallel group randomized trials [13], and the checklist from the guideline for RSA implant migration measurements was used [14].

Patients were block-randomized (2 or 4 cases in each block) and assigned, in a 1:1 ratio, to either robotically assisted or conventional TKA, to ensure equal numbers of each method per surgeon. The randomized lists for each surgeon were created according to software randomization and kept at a separate location from the operating department. Surgeons called a research assistant before surgery and were informed of the assigned method (conventional or robotic).

The patients and observers (physiotherapist and radiologists) were blinded to which surgical method was used. 60 patients were enrolled in the RSA analysis, where 36 (18 in each arm) had complete data sets and 51 had index plus 1 or more image(s) ready for analysis.

### Setting

Patients were recruited and treated at Haugesund Hospital for Rheumatic Diseases (HSR) in Norway by 2 experienced arthroplasty surgeons (22 and 29 operations each in the study). The surgeons performed 10 robotically assisted TKAs, as well as cadaver operations prior to patient inclusion in the trial.

### Patients

Inclusion criteria were patients with an age from 45–85 years old, with primary or secondary osteoarthritis (OA).

Exclusion criteria included active infection, degenerative neuromuscular disease, active metastasizing cancer, severe cardio- or pulmonary disease, ASA class 4, dementia, serious systemic disease, and previous fracture or deformity making use of intramedullary rods impossible.

### Intervention

The bi-cruciate stabilized Journey II BCS total knee replacement (Smith & Nephew, Memphis, TN, USA) was implanted in all knees using Palacos R+G cement (Heraeus, Hanau, Germany). The patella was resurfaced, and the lower limit for remaining patellar thickness was 12 mm. In the conventional group the measured resection technique [15] with a default of 3° tibial posterior slope, and 5–7° valgus on the distal femoral cut, aiming for a neutral mechanical alignment, was used. For the robotic group (NAVIO system, Smith & Nephew, Memphis, TN, USA) we used a functional alignment technique [16], starting with components in mechanical overall alignment (software default), then shape-matching to the bony and cartilaginous structures, and finally adjusting the gaps in flexion and extension, to comply with good ligament balance, restricted to 2° valgus and 4° varus. If ligament balance was not met within these restrictions, soft tissue release was performed. Tibial posterior slope was rarely altered from the system default of 3°. A hand-held robotic burr was used for the distal femoral cut and for holes supporting the tibia cutting guide, which was secured in place by additional speed pins. The cutting guide position was checked with the navigation system before cuts were performed. The cut surfaces were in some cases refined with a burr, for better accuracy and component fitting. Conventional operations were all performed with intramedullary rods for both tibia and femur. A torniquet was used for all procedures, as well as a 1-phase cementing technique, after pulsed lavage. Tranexamic acid i.v. injection 1.5 g was administered 10 minutes before surgery for all patients. No drains were used. Anti-thrombotic medication (Fragmin 5,000 U) was administered as a subcutaneous injection 10 hours after surgery and continued for 14 days (self-administration). Antibiotic prophylaxis (cefazoline 2 g i.v. injection) was administered 30–20 minutes before surgery, then after 4, 8, and 12 hours. The skin incision was closed with running mattress sutures. The operated knee was positioned in 90° flexion for 2 hours post-surgery to reduce hematoma. The patients were mobilized and allowed full weightbearing after surgery and trained with standardized exercises from the first postoperative day.

### RSA

For the RSA analysis, 6 tantalum markers (diameter 0.8 and 1.0 mm) were inserted during surgery into the polyethylene through slightly undersized drilled tunnels. In the tibial metaphysis, 9 tantalum markers (1.0 mm) were inserted according to a defined protocol, before the tibial component was implanted. No additional incision was used in this study as the reflector disc pins were drilled bi-cortically into bone within the primary incision in both femur and tibia, extending the incision slightly in some of the robotic cases.

The index RSA radiographs were taken on day 1, 2, or 3 (conventional mean 1.7 days, SD 0.5, robotic mean 2.0 days, SD 0.5) after weightbearing, and repeated at 3, 12 (conventional mean 386 days, SD 18, robotic mean 386 days, SD 22), and 24 months after surgery. The patients were supine with the knee positioned inside a biplane calibration cage (cage 10; RSA Biomedical, Umeå, Sweden) according to a technique described earlier [17]. 1 portable X-ray tube and 1 gantry-mounted X-ray tube were used to obtain 2 simultaneous exposures at a 90° angle. For radiographic imaging, we used high-definition digital plates (Agfa CR MD 4.0) and for plate reading we used the ADC compact digitizer (Agfa, Mortsel, Belgium).

The investigators involved in the RSA examinations and analysis were blinded to the patient allocation. However, the fixator pin holes securing the reflector discs could be seen on some of the index images from the robotic group. The RSA measurements were possible if 3 or more of the markers were visible in each segment for repeated examinations. Translation and rotation of the tibial component relative to bone was calculated using the markers in the polyethylene insert as the moving segment and tibia as the fixed reference segments (UmRSA Digital Measure version 6.0; RSA Biomedical, Chatham, Kent, UK).

The precision of the RSA measurements was calculated after double examinations at 12 months and showed a mean difference of maximum total point motion (MTPM) of 0.13 mm (CI 0.10–0.17) in the conventional group and 0.14 mm (CI 0.10–0.18) in the robotic group.

Mean and SD of number of markers, condition number, and mean error of body fitting for each rigid body at the primary follow-up timepoint were 5.72 (0.54), 25.4 (5.31), and 0.08 (0.16) respectively.

### Outcomes

The movements of the implant were measured along and around a medially directed axis (x-axis, anteroposterior rotation [AP]), sagittal axis (z-axis, varus–valgus rotation), and longitudinal axis (y-axis, internal–external rotation) of the knee. The condition number was set at 150, and the upper limit for mean error of body fitting at 0.35 to ensure proper stability and distribution of the tantalum markers [17,18]. Translations were expressed as MTPM, subsidence, and lift-off. MTPM represents the 3-D vector of the prosthetic marker that moved the most and is a measure of the magnitude of migration only [19]. The largest positive Y-translation was called lift-off and the largest negative Y-translation was called subsidence. The calculations were performed according to the orthogonal right-handed coordinate system.

The primary outcome for this RSA analysis was MTPM of the tibial components at 1 and 2 years after surgery with robotic and conventional technique.

The secondary outcomes were x-, y-, and z rotation, subsidence, and lift-off of the tibial component.

### Statistics

A power analysis previously used in a similar study [20] was used to determine the number of patients included in this present analysis, claiming 0.1 mm as a clinically relevant between-group difference, and with an anticipated repeatability of 0.1 mm in the RSA measurements. A group sample size of 17 would achieve 80% power to detect a difference of 0.1 between groups with an estimated standard deviation of 0.1 and with a significance level of (alfa) 0.05, using a 2-sided, 2-sample t-test. To ensure proper sample size at 2 and 5 years we chose to include 60 patients in the study, 30 in each group.

To evaluate the RSA measurement precision, the difference between double measurements was calculated after 1 year. We also calculated the standard deviation (SD) of the differences with respect to zero [21].

Magnitude and progress of migration are presented as mean values, whereas for the difference between groups the median values are used, and only absolute values were analyzed. The Mann–Whitney test was used to compare the conventional and robotic groups for migration. The Shapiro–Wilk and Pearson test showed that migration data was not normally distributed, so both median and interquartile range (IQR) are reported. Pearson’s chi-square test was used to assess the differences in age, sex, and diagnosis. For statistical analysis, the median difference and the corresponding CI for the median difference for each migration and clinical parameter were calculated as described by Campbell and Gardner (1988)[22]. P value < 0.05 was considered significant. Statistical evaluation was performed using Stata version 18 (StataCorp LLC, College Station, TX, USA).

### Ethics, registration, data sharing plan, funding, use of AI, and disclosures

The study was registered in the trial database at ClinicalTrials.gov (identifier: NCT04525950) on September 7, 2020. The trial was approved by the regional committee for medical and health research ethics (REK), Bergen, Norway on March 4, 2020 (ref.no.68448 NAVIO HSR). Haugesund Sanitetsforenings Forskningsfond gave financial support. AI tools were not used at any stage in this submission, and no author had any conflict of interest. Data is available by contacting the corresponding author.

Complete disclosure of interest forms according to ICMJE are available on the article page, doi: 10.2340/17453674.2025.43081

## Results

**Figure 1:**
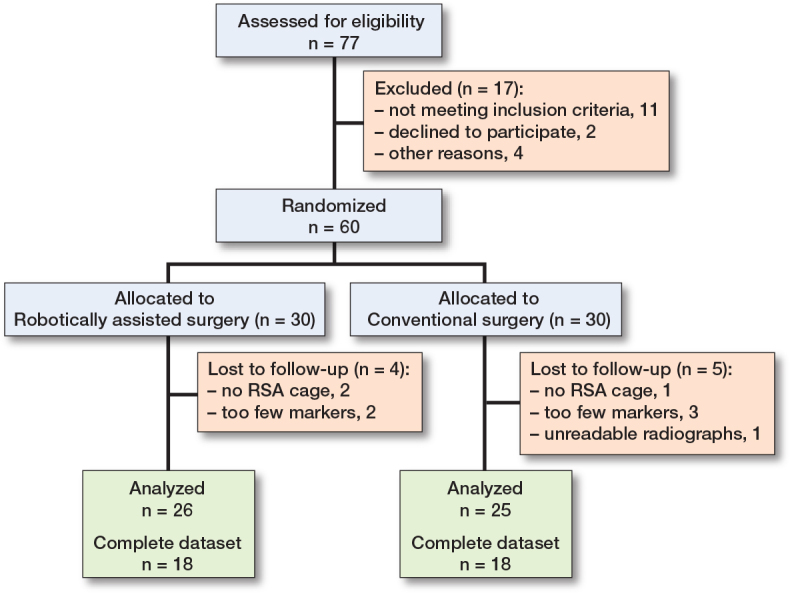
Flowchart of patients included in the study.

We enrolled 60 patients between September 2020 and June 2021 where 55 were sufficiently tantalum marked, excluding 3 patients with no RSA cage, 1 with no image taken on day 2–3 after surgery, leaving 51 for analysis, 26 in the robotic and 25 in the conventional group. Some of the patients did not have a full data set, for various reasons, but were included if at least 2 RSA examinations, including the index examination, were performed (Figure 1) and omitted from those time points where no images were captured. The last follow-up images were captured in June 2023.

1 of the included patients died before the 2-year examination.

The operating time was 103 minutes (SD 19) in the robotic group and 88 minutes (SD 18) minutes in the conventional group (Table 1).

**Table 1:** Patient characteristics and operating time. Data presented as count or mean (SD)

Factor	Conventional n = 25	Robotic n = 26
Men	10	14
Mean age (SD)	67.7 (3.9)	65.7 (3.7)
ASA class		
1	1	4
2	20	16
3	3	6
Missing	1	–
Diagnosis		
Idiopathic osteoarthritis	18	19
Other	7	7
Mean operating time, minutes (SD)	88 (17)	103 (19)

### Outcome

**Figure 2:**
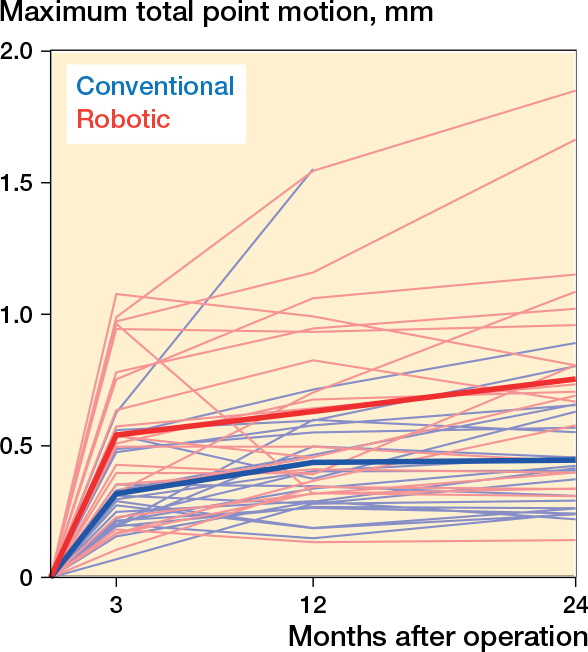
Spaghetti plot with individual and mean maximum total point motion.

The tibial component migrated most during the first 3 months for both groups and tapered off thereafter (Figure 2). The mean MTPM up to 12 and 24 months was higher in the robotic: 0.64 mm (CI 0.49–0.78) and 0.75 mm (CI 0.57–0.92), than in the conventional group: 0.44 mm (CI 0.33–0.56) and 0.46 mm (CI 0.37–0.54), respectively (Table 2). The median difference in MTPM between the robotic and conventional group was 0.14 mm (CI 0.02–0.35; P = 0.03) and 0.21 mm (CI 0.05– 0.44; P = 0.01) at 12 and 24 months respectively (Table 3).

**Table 2:** Migration up to 24 months for the conventional and the robotic group

Factor Months FU	Conventional		Robotic	
	Median [IQR]	Mean (CI)	Median [IQR]	Mean (CI)
X-axis rotation [flexion–extension] (°, absolute value)				
3	0.09 [0.05–0.22]	0.14 (0.09–0.20)	0.12 [0.07–0.26]	0.20 (0.12–0.27)
12	0.14 [0.06–0.26]	0.21 (0.12–0.29)	0.32 [0.10–0.41]	0.30 (0.20–0.39)
24	0.15 [0.05–0.32]	0.20 (0.13–0.27)	0.36 [0.14–0.55]	0.39 (0.25–0.53)
Y-axis rotation [internal–external] (°, absolute value)				
3	0.09 [0.04–0.20]	0.13 (0.08–0.19)	0.11 [0.08–0.19]	0.14 (0.09–0.20)
12	0.13 [0.08–0.24]	0.22 (0.07–0.37)	0.23 [0.08–0.33]	0.24 (0.16–0.31)
24	0.14 [0.09–0.25]	0.19 (0.12–0.26	0.27 [0.07–0.43]	0.30 (0.19–0.42)
Z-axis rotation [varus–valgus] (°, absolute value)				
3	0.09 [0.05–0.14]	0.10 (0.07–0.13)	0.09 [0.06–0.26]	0.23 (0.10–0.35)
12	0.16 [0.06–0.26]	0.17 (0.11–0.24)	0.11 [0.06–0.27]	0.24 (0.11–0.36)
24	0.13 [0.07–0.21]	0.17 (0.09–0.24)	0.10 [0.05–0.20]	0.24 (0.10–0.37)
Maximum total point motion, mm				
3	0.29 [0.20–0.49]	0.35 (0.27–0.44)	0.47 [0.28–0.77]	0.52 (0.40–0.65)
12	0.39 [0.28–0.55]	0.44 (0.33–0.56)	0.48 [0.38–0.94]	0.64 (0.49–0.78)
24	0.42 [0.26–0.63]	0.46 (0.37–0.54)	0.70 [0.40–0.99]	0.75 (0.57–0.92)
12–24 **^a^**	0.16 [0.12–0.20]	0.16 (0.14–0.19)	0.19 [0.14–0.26]	0.23 (0.16–0.29)
Maximum lift-off, mm				
3	0.09 [0.05–0.13]	0.09 (0.06–0.12)	0.13 [0.04–0.24]	0.21 (0.11–0.30)
12	0.11 [0.07–0.24]	0.14 (0.09–0.19)	0.18 [0.11–0.33]	0.25 (0.15–0.36)
24	0.13 [0.05–0.22]	0.15 (0.10–0.21)	0.15 [0.10–0.42]	0.28 (0.17–0.40)
Maximum subsidence, mm				
3	0.08 [0.05–0.12]	0.10 (0.07–0.14)	0.12 [0.06–0.16]	0.14 (0.08–0.20)
12	0.09 [0.06–0.17]	0.13 (0.07–0.19)	0.13 [0.04–0.23]	0.17 (0.05–0.29)
24	0.11 [0.04–0,18]	0.13 (0.07–0.20)	0.14 [0.04–0.28]	0.21 (0.06–0.36)

a Migration between 12 and 24 months for conventional and robotic

IQR = Interquartile range; CI = 95% confidence interval; FU= follow-up.

**Table 3:** Median difference in migration between robotic and conventional cemented tibial components up to 24 months, and between 12 and 24 months

Factor	Conventional median	Robotic median	Median difference (CI)	P value
Conventional vs robotic at 12 months				
X rotation	0.14	0.32	–0.09 (–0.22 to 0.02)	0.1
Y rotation	0.13	0.23	–0.05 (–0.15 to 0.03)	0.3
Z rotation	0.16	0.11	0.01 (–0.06 to 0.09)	0.7
MTPM	0.42	0.70	–0.14 (–0.35 to –0.02)	0.03
Lift-off	0.11	0.18	–0.06 (–0.14 to 0.03)	0.1
Subsidence	0.09	0.13	0.01 (–0.06 to 0.09)	0.7
Conventional vs robotic at 24 months				
X rotation	0.15	0.36	–0.14 (–0.33 to –0.00)	0.049
Y rotation	0.14	0.27	–0.07 (–0.23 to 0.04)	0.3
Z rotation	0.13	0.11	0.00 (–0.07 to 0.06)	0.99
MTPM	0.42	0.70	–0.21 (–0.44 to –0.05)	0.01
Lift-off	0.13	0.15	–0.06 (–0.18 to 0.01)	0.1
Subsidence	0.11	0.14	0.03 (–0.06 to 0.12)	0.5
Conventional vs robotic from 12–24 months				
MTPM	0.16	0.19	–0.03 (–0.08 to 0.01)	0.1
Lift-off			–0.02 (–0.06 to 0.01)	0.3
Subsidence			0.01 (–0.04 to 0.03)	0.8

CI = 95% confidence interval; MTPM = maximum total point motion.

No difference was found between the groups for the median migration between 12 and 24 months, 0.03 mm (CI –0.01 to 0.08; P = 0.1) (Table 3).

The overall mean MTPM migration up to 12 months for all patients was 0.54 mm (CI 0.44–0.63) and the overall mean MTPM migration between 12 and 24 months was 0.19 mm (CI 0.16–0.23).

The median difference between the groups for flexion/extension migration of the tibia at 24 months was 0.14° (CI 0.00–0.33), P = 0.049 (Table 3).

There were no significant differences between the groups for maximum subsidence and lift-off at 12 and 24 months.

## Discussion

This is the first study to compare implant migration in a robotically assisted computer-navigated technique with conventional TKAs. The use of robotic assistance for the implantation of the bi-cruciate stabilized prosthesis was associated with an increased migration of the tibial component occurring within the observation period. However, the migration of the robotic group tapered off, and did not migrate more than the conventional group between 12 and 24 months. The overall migration for the bi-cruciate stabilized implant, regardless of operating technique, was within acceptable limits. There were 4 patients with MTPM migration at 12 months above 1.0 mm in the robotic group, the highest being 1.5 mm, whereas in the conventional group only 1 patient was above 1.0 mm (1.5 mm).

The mean MTPM migration from 12 to 24 months was not significantly different between the groups, measuring < 0.2 mm in the conventional and just above 0.2 mm in the robotic group. In a previous study by Ryd et al., the limit for strict continuous migration (SCM) was set at 0.2 mm after 12 months, above which the risk of aseptic loosening was increased [19]. The overall migration up to 12 months was 0.54 mm and 0.19 mm from 12–24 months. Pijls et al. suggested a limit for unacceptable migration with a high rate of early aseptic loosening at 1.6 mm at 12 months, and under 0.5 mm was considered the limit under which all studies showed acceptable survival of the prostheses [23]. These thresholds were reaffirmed in a recent updated systematic review [24]. In the light of these previous studies, we conclude that the overall migration in the present study was within acceptable limits. A previous randomized clinical RSA study comparing conventional with navigated cemented Profix CR TKAs [20] as well as a recent cohort study with a new ball-in-socket (BS) medial conformity CR TKA [25] had lower MTPM migration values up to 24 months than the values measured in our study, while another RSA study comparing the results of a symmetrical cemented PS (NexGen, Zimmer Biomet) to an asymmetrical cemented PS (Persona, Zimmer Biomet, Warsaw, IN, USA) TKA, had higher MTPM migration values up to 24 months [26]. A recent RSA cohort study shows a higher risk of aseptic loosening for PS compared with CR TKAs [27]. The migration in the robotic group was higher than in the conventional group in our study, but lower than the migration patterns of both tibial components in the Nexgen-Persona study.

### Strengths

The main strength of this analysis is the RCT design comparing migration between robotic and conventional TKA alignment techniques. The number of patients was sufficient to evaluate whether there was a statistically and clinically significant difference in implant migration between the groups [26,28]. 2 experienced arthroplasty surgeons from the same department (ØS and ØG) operated on every case together; consequently, there could be only small variations regarding the surgery.

### Limitation

The main limitation was that the number of participants was too small to evaluate subgroups (age groups and implant sizes) within the study population. The study did not evaluate the migration of the femoral component. Only the tibial component migration was investigated, as aseptic loosening is more common on the tibial side.

The patients in the robotic group had bi-cortical metaphyseal pin holes in the tibia for the reflector disc assembly, more distal tibial cutting guide fixation, no opening of the tibial intramedullary canal, different alignment technique, and a higher degree of femoral component flexion (4° vs 2°). The alignment target differed between the groups and could theoretically lead to more shear forces and changes in migration.

### Conclusion

The cemented bi-cruciate stabilized total knee implant migrates more in the first 2 years, when operated with robotic assistance, but after year 1 the migration pattern did not differ for the 2 techniques. However, the overall migration pattern is considered within safe limits.

## Notes

ØS: designed the study, acquired data and wrote the manuscript. ØG: co-designed the study, analyzed data, and reviewed the manuscript. OF: co-designed the study and reviewed the manuscript. SHLL: analyzed the data, reviewed the manuscript, and contributed to figures and tables. IOM: produced the RSA data and reviewed the manuscript. GH, PJH: reviewed the manuscript. AMF: analyzed the data and reviewed the manuscript.

The authors would like to thank radiographers at Haugesund Hospital, Helse Fonna, for producing high-quality RSA images, and coordinator Beathe Angstaur for organizing all the patient follow-ups.

They also would like to thank the surgical team and staff at the Orthopaedic Department of Haugesund Hospital for Rheumatic diseases (RKO) for all support and help with this study, and Haugesund Sanitetsforenings Forskningsfond for the financial support.

They thank Randi Furnes for help with the randomization of patients.

Handling co-editors: Keijo Makela and Robin Christensen

*Acta* thanks Poul Torben Nielsen and Olof Sandberg for help with peer review of this manuscript.
